# Susceptibility to Intracellular Infections: Contributions of TNF to Immune Defense

**DOI:** 10.3389/fmicb.2020.01643

**Published:** 2020-07-15

**Authors:** Xinying Li, Heinrich Körner, Xiaoying Liu

**Affiliations:** ^1^Translational Research Institute, Academy of Medical Science, Henan Provincial People’s Hospital, Zhengzhou, China; ^2^School of Life Sciences, Anhui Medical University, Hefei, China; ^3^Key Laboratory of Anti-inflammatory and Immunopharmacology, Institute of Clinical Pharmacology, Ministry of Education, Engineering Technology Research Center of Anti-inflammatory and Immunodrugs in Anhui Province, Anhui Medical University, Hefei, China

**Keywords:** TNF, macrophage polarization, *Listeria monocytogenes*, *Mycobacterium tuberculosis*, *Leishmania major*

## Abstract

An interesting puzzle is the fact that an infection of a tumor necrosis factor α (TNF)-deficient host with pathogens such as bacteria or parasites that reside intracellularly inevitably ends fatally. Is this due to one specific role of TNF in the immune defense or are different functions responsible for this outcome? In this review we provide an update of the functions of TNF in the defense against the intracellular pathogens *Listeria monocytogenes*, *Mycobacterium tuberculosis*, and *Leishmania major*. Furthermore, we discuss the role of TNF in the generation of proinflammatory macrophages in mouse models of infection and summarize briefly the potential consequences of anti-TNF treatment for infectious diseases.

## Introduction

The increased susceptibility of a host organism to pathogens has many underlying factors. These range from environmental causes such as the effects of malnourishment on the immune system through to intrinsic differences resulting from genetics. Some susceptibility genes have been relatively easy to identify since the functions of their encoded proteins are directly associated with pathogenic resistance. For example, the natural resistance-associated macrophage protein (Nramp) has been shown to be crucial in the fight of macrophages against intracellular pathogens including mycobacteria, salmonella, and leishmania (Vidal et al., 1993). Susceptibilities based on deficiency or dysregulation of cytokines are usually more complex because these can originate from direct or indirect activities, or secondary interactions with the host immune system. In this respect, the cytokine tumor necrosis factor α (TNF) is an excellent example which has been studied extensively because of its involvement in susceptibilities to pathogens. This cytokine was named for its ability to induce haemorrhagic necrosis in murine Meth A sarcomas (Carswell et al., 1975). Macrophages activated by endotoxin were identified as the major source of TNF (Mannel et al., 1980) with natural killer cells and T lymphocytes constituting the predominant secondary sources in response to various challenges (Fremond et al., 2005). TNF plays a central role in initiation and promotion of inflammation (Old, 1985; Körner and Sedgwick, 1996; Sedgwick et al., 2000), thus dysregulated TNF production and function are associated with the pathogenesis of inflammatory diseases such as rheumatoid arthritis (RA), inflammatory bowel disease, systemic lupus erythematosus, ankylosing spondylitis, and multiple sclerosis (Kollias et al., 1999; Kollias, 2005). To date, biologicals which effectively neutralize TNF activity have not shown utility for multiple sclerosis, but have had varying degrees of efficacy for other conditions. The antagonists include anti-TNF monoclonal antibodies and an anti-TNF receptor-Fc fusion protein. Unfortunately, due to the prominent pro-inflammatory function of TNF, administration of anti-TNF agents is associated with an increased risk of an infection, especially by intracellular pathogens (Efimov et al., 2009) and malignancies (Bongartz et al., 2006). In this review, we will focus on the role of TNF in intracellular infections by bacterial and parasitic pathogens such as *Listeria monocytogenes*, *Mycobacterium tuberculosis*, and *Leishmania* spp. Since macrophages are the predominant host cells of many of those infections, this cell type and the role of TNF in its differentiation will be discussed in particular.

## Effects of TNF in *Listeria monocytogenes* Infection

*Listeria monocytogenes* is an intracellular, food-borne, gram-positive pathogen that causes listeriosis, which is characterized by gastroenteritis, septicaemia, and meningitis. Infections have high hospitalization and fatality rates in immunocompromised patients and can result in spontaneous abortions during pregnancy (Gellin and Broome, 1989; Schuchat et al., 1991). After ingestion, *L. monocytogenes* invades the blood stream and lymphatic system, and ultimately disseminates to spleen, liver and potentially, the central nervous system (Schuchat et al., 1991). In this context, *L. monocytogenes* has emerged as a focal point of research into genetics and strategies of intracellular pathogens (Glaser et al., 2001). Whilst it can infect non-phagocytic cells, including hepatocytes, enterocytes, endothelial cells, and fibroblasts, and can also be internalized into phagocytes such as macrophages through phagocytosis (Vazquez-Boland et al., 2001). Once internalized, the bacterium may be killed or alternatively can escape from the phagolysosome into the cytoplasm and initialize infection of other host cells (Cossart and Mengaud, 1989; Tilney and Portnoy, 1989).

A control of *L. monocytogenes* infection requires an activation of toll-like receptors (TLRs) and a functional MyD88 pathway which leads to the expression of proinflammatory cytokines, including TNF and IL-12 (Tripp et al., 1993). The pathogen also activates AIM2, NLRP3, NLRC4, NLRC5, NLRC6 inflammasomes and induces the release of IL-1β and IL-18 (Theisen and Sauer, 2016). The initial boost of these pro-inflammatory cytokines is important because it triggers IFN-γ secretion by T cells which has been shown to be crucial for macrophage activation and microbicidal activity (Buchmeier and Schreiber, 1985; Dalton et al., 1993). TNF contributes to protection against infection in an experimental model (Havell, 1987) and acts as co-stimulator for the crucial production of IFN-γ (Tripp et al., 1993). It is also involved in a T cell-independent pathway that leads to macrophage activation as demonstrated in infection of severe combined immunodeficiency mice (Bancroft et al., 1989). Moreover, the contribution of TNF was shown in gene-deficient mouse models where either TNF or TNF Receptor 1 (R1)-deficient mice quickly succumbed to *L. monocytogenes* rather than recovering after a few days as occurs in control mice (Pfeffer et al., 1993; Rothe et al., 1993; Pasparakis et al., 1996). TNF is generated as a membrane protein before cleavage and release and notably, knock-in of an uncleavable form of TNF controlled *L. monocytogenes* infection at a low dose suggesting membrane bound TNF in macrophages functions to some extent in anti-bacterial responses (Torres et al., 2005; Table 1).

**TABLE 1:** Consequences of interference with TNF signaling in *L. monocytogenes* infection.

**Pathogen**	**Genetic background/treatment**	**Infection site**	**Phenotype**	**References**
*L. monocytogenes*	C57BL/6-TNF^–/–^	IP	Susceptible, increased bacterial load in spleen, liver	Pasparakis et al., 1996; Li et al., 2017
	C57BL/6-MN-TNF^–/–^	IV	Susceptible to low-mediate dose of infection	Grivennikov et al., 2005
	C57BL/6-T cells-TNF^–/–^	IV	Susceptible to high dose of infection	Grivennikov et al., 2005
	C57BL/6-TNFR1^–/–^	IV	Susceptible, increased bacterial load in spleen, liver	Pfeffer et al., 1993; Rothe et al., 1993
	C57BL/6-TNFR2^–/–^	IV	Susceptible to high dose of infection, normal T cell development	Erickson et al., 1994
	rHuTNF	IV	Resistant to lethal bacterial infection	Desiderio et al., 1989

*IP, intraperitoneal; IV, intravenous; TNF, tumor necrosis factor α; MN, macrophages/neutrophils; rHuTNF, recombinant human TNF.*

Human RA patients that were treated with anti-TNF biologicals had an increased general infection rate (Listing et al., 2005). This was confirmed in a meta-analysis of the literature (Bongartz et al., 2006) which has been discussed controversially (Dixon and Silman, 2006). In a second national prospective observational study of 7,664 anti-TNF-treated patients that used data from the British Society for Rheumatology Biologics Register with severe RA, 19 serious infections with intracellular pathogens were isolated without a preference for either infliximab or adalimumab (Dixon et al., 2006).

More specifically, serious infections with *L. monocytogenes* after treatment with either infliximab or etanercept were identified in the FDA Adverse Event Reporting system in a small number of patients. In 14 of the total 15 cases were associated with infliximab treatment (Slifman et al., 2003). In a more recent study that used the same source from 2004 to 2011, 266 cases of listeriosis could be identified with the majority of patients receiving infliximab (77.1%), followed by etanercept (11.7%), and adalimumab (9.8%) (Bodro and Paterson, 2013). The differences between infliximab and etanercept activity are interesting conundrums and are most likely due to the different modes of action between the two biologicals (Fallahi-Sichani et al., 2012). Infliximab is chimeric monoclonal antibody fused with human IgG1 targeting TNF while etanercept is a fusion protein with a human IgG1 Fc tail and TNFR2, but only infliximab causes monocytopenia (Lugering et al., 2001) and can induce apoptosis in intestinal T cells (Van Den Brande et al., 2003).

## TNF in *M. tuberculosis* Infection

The infectious disease tuberculosis (TB) is caused by an infection with *M. tuberculosis* and constitutes a leading cause of mortality worldwide (Kaufmann et al., 2005). With multidrug resistant strains spreading, and in combination with HIV infection, tuberculosis is a global health threat (Kaufmann, 2001). Predominantly, healthy individuals exposed to *M. tuberculosis* will not develop clinical disease symptoms. Only 10% of infections eventually show clinical signs of tuberculosis (O’garra et al., 2013). In the lungs, neutrophils and alveolar macrophages are crucial in initiating an early inflammatory response (Seiler et al., 2003). This early response is essential for the formation of an heterogeneous granuloma that is composed of macrophages, dendritic cells, neutrophils, NK cells, B and T cells, and contains the bacteria (Guirado and Schlesinger, 2013).

Neutralization of TNF or TNFR1-deficiency due to a genetic modification in mouse models results in high susceptibility to *M. tuberculosis* infection with increased bacterial loads (Flynn et al., 1995). This increase in susceptibility is exclusively driven by the soluble TNF-TNFR1 axis (Flynn et al., 1995). In contrast, membrane-bound TNF and TNFR2 play minor roles in anti-mycobacterial defense (Jacobs et al., 2000; Olleros et al., 2002).

Surprisingly, the underlying cause for the increased sensitivity of TNF-deficient mice is the lack of organized granulomas. Despite an accumulation of lymphocytes in the perivascular areas of the lung, they fail to collocate with inflammatory macrophages at the site of *M. tuberculosis* infection (Bean et al., 1999). Indeed, at the late stage of infection of TNFRp55^–/–^ mice, a lethal disintegration of the poorly formed granulomas occurs (Ehlers et al., 2000; Table 2).

**TABLE 2:** Consequences of interference with TNF signaling in *M. tuberculosis* infection.

**Pathogen**	**Genetic background/treatment**	**Infection site**	**Phenotype**	**References**
*M. tuberculosis*	C57BL/6-TNF^–/–^	IV	Susceptible, delayed granuloma formation in liver	Bean et al., 1999
	C57BL/6-memTNF^–/–^	Aerosol	Resistant to low dose of bacterial infection	Saunders et al., 2005
	C57BL/6-TNFR1^–/–^	IV	Susceptible, high bacterial load in spleen, liver, lungs, delayed granuloma formation	Flynn et al., 1995
	C57BL/6-TNFR2^–/–^	IV	Less susceptible, normal granuloma formation	Jacobs et al., 2000
	Anti-TNF mAb	IV	Susceptible, high bacterial load in spleen, liver, lungs	Flynn et al., 1995
	Anti-TNF mAb in quiescent phase	IV	Reactivation of infection, disorganization of granuloma	Mohan et al., 2001

*IV, intravenous; TNF, tumor necrosis factor α; mAb, monoclonal antibody; memTNF, membrane TNF.*

Along with cytokines such as TNF, inducible nitric oxide synthase (iNOS) has also been produced as an important effector in host defense against *M. tuberculosis*. A role of iNOS was first shown using inhibitors of iNOS which increased the bacterial burden and also the tissue injury of *M. tuberculosis* infection (Chan et al., 1995). Moreover, findings in genetically deficient iNOS^–/–^ mice confirming iNOS was an important host gene locus for susceptibility (Macmicking et al., 1997). Interestingly, experiments in TNF^–/–^ mice indicated that the role of iNOS in resistance was not as dominant as had been suggested. Despite TNF-deficient mice failing to establish protective granulomas and rapidly succumbing to *M. tuberculosis* infection, they were able to produce normal amounts of iNOS (Bean et al., 1999). The view that iNOS plays a limited role in protection was supported in experiments comparing IFN-γ-deficient mice to iNOS^–/–^ mice. In the early stage of a low dose infection model, IFN-γ expression was crucial in controlling disease, whereas iNOS^–/–^ mice controlled *M. tuberculosis* infection almost as effectively as wild-type mice (Cooper et al., 2000). A caveat of these findings involves different experimental conditions, in particular, differences in routes of infection, strains of pathogen and analysis time points. Furthermore, at the later stages of infection, IFN-γ-deficient mice succumbed to disease and the reduced iNOS expression in macrophage suggests that iNOS was important to recovery.

While host susceptibility is important, another key factor determining the severity of infections is bacterial virulence. Experiments with virulent (H37Rv) and attenuated (H37Ra) *M. tuberculosis* strains in resistant and iNOS^–/–^ mouse strains showed that the role of iNOS in protection depended to a large extent on the virulence of the infecting strain. The attenuated strain was controlled by both mouse strains while the iNOS^–/–^ mice showed exacerbated infection with the virulent strain (Beisiegel et al., 2009).

This limited role of iNOS as protective factor has also been shown in reactivation experiments. CD4^+^ T cell depletion caused reactivation despite unimpaired iNOS expression implying other macrophage factors in host defense (Scanga et al., 2000) and neutralization of TNF after successful treatment that reduced the number of pathogens to an undetectable level resulted in a rapid reactivation (Botha and Ryffel, 2003).

About one quarter to one third of the human population carries a latent *M. tuberculosis* infection that is encapsulated in granulomas and can stay dormant for the entire life span of the carrier (Getahun et al., 2015). Reactivation of tuberculosis in patients due to anti-TNF treatment has been recognized as a major complication (Keane et al., 2001) and it became clear that a difference existed between treatment with antibody- or receptor-based biologicals (Ehlers, 2005). While the underlying mechanisms are still not entirely clear, computer modeling shows that binding to membrane TNF is critical for an impairment of granuloma formation and that the differences between drugs in binding kinetics and vascular permeability are crucial (Fallahi-Sichani et al., 2012).

## TNF in Cutaneous Leishmaniasis

The parasite *Leishmania* spp. causes cutaneous leishmaniasis which is common in South America, Sub-Saharan Africa, the Mediterranean, the Middle-East and parts of South-East Asia with about one million new cases annually (Reithinger et al., 2007; Ready, 2014). Leishmaniasis has been identified as a neglected tropical disease by the world health organization and lacks an effective vaccine (Kaye and Aebischer, 2011). The transfer of *Leishmania* spp. promastigotes into the skin occurs during a blood meal of female phlebotomine sand flies (Sacks and Perkins, 1984). Cutaneous leishmaniasis is the most common form of leishmaniasis with benign self-healing skin lesions that eventually resolve, while muco-cutaneous leishmaniasis leads to complications that result in permanent disfigurement of the patients. Finally, the visceral leishmaniasis represents the most severe form of leishmaniasis and presents with fever, serious weight loss, hypergammaglobulinemia, and pancytopenia (Reithinger et al., 2007).

*Leishmania* spp. parasites initially encounter neutrophils in the skin and are phagocytosed (Laskay et al., 2003; Van Zandbergen et al., 2004; Peters et al., 2008). After apoptosis, the parasitized neutrophils are then taken up by macrophages (Pearson et al., 1983; Van Zandbergen et al., 2004). Amastigotes can find shelter from an immune response and multiply inside the macrophage phagolysosome while tapping into the macrophage metabolism for nutrients (Bogdan et al., 1996; Bogdan and Rollinghoff, 1998).

However, once the pro-inflammatory cytokines IFN-γ and TNF become available, macrophages begin producing microbicidal effector molecules such as iNOS and can eliminate the contained pathogens (Bogdan et al., 1990; Green et al., 1990; Liew et al., 1990a; Hu et al., 2017).

The role of TNF in defense against *Leishmania major* has been intensively investigated in experimental cutaneous leishmaniasis *in vivo* models using mice deficient in TNF ligand or TNF receptors. Deficiency of soluble TNF leads to a high parasite burden, unresolved lesions and fatal disease, despite a Th1-response (Wilhelm et al., 2001; Fromm et al., 2015; Hu et al., 2017). Counterintuitively, TNFR1-deficient mice show an elevated parasite load but eventually resolve lesions and eliminate the *L. major* parasites (Vieira et al., 1996; Fromm et al., 2015). Moreover, TNFR2-deficient (Nashleanas et al., 1998; Fromm et al., 2015) and membrane TNF-deficient (Allenbach et al., 2008; Fromm et al., 2015) are resistant to *L. major* infection, indicating that soluble but not membrane TNF plays the key role in the defense against *L. major* (Table 3).

**TABLE 3:** Consequences of interference with TNF signaling in *L. major* infection.

**Pathogen**	**Genetic background/treatment**	**Infection site**	**Phenotype**	**References**
*L. major*	C57BL/6-TNF^–/–^ (B6.TNF^–/–^)	SC	Extremely susceptible, high parasite burden, fail to resolve lesions, produce iNOS but no NO; higher ratio of iNOS^+^Arg1^+^ versus iNOS^+^ macrophages	Körner et al., 1997; Fromm et al., 2012; Schleicher et al., 2016; Hu et al., 2018
	C57BL/6-memTNF^Δ/Δ^	SC	Resistant, similar to wild-type	Allenbach et al., 2008; Fromm et al., 2012
	C57BL/6 TNFR1^–/–^	SC	Susceptible, high parasite burden, fail to resolve lesions, survive infection in many cases	Vieira et al., 1996; Nashleanas et al., 1998; Fromm et al., 2012
	C57BL/6-TNFR2^–/–^, TNFR1/R2^–/–^	SC	Resistant, similar to wild-type	Nashleanas et al., 1998; Fromm et al., 2012
	rTNF	SC	Smaller lesion size, lower parasite counts	Titus et al., 1989; Liew et al., 1991

*TNF, tumor necrosis factor α; memTNF, membrane TNF; rTNF, recombinant TNF; SC, subcutaneous.*

The expression of iNOS after activation by IFNγ and TNF has been shown to be central for an effective host response (Bogdan et al., 1990; Green et al., 1990; Stenger et al., 1994). In the early stages after parasite inoculation, iNOS expression is induced by IFNα/β signaling in the skin to prevent the dissemination of parasites (Diefenbach et al., 1998). IFN-γ expression by CD4^+^ T cells also promotes the expression of iNOS (Stenger et al., 1994) which generates a high NO concentration in the tissue and effectively controls *Leishmania* expansion (Olekhnovitch et al., 2014). After clinical resolution of the lesions, the disease will reappear and *Leishmania* proliferate rapidly if iNOS activity is inhibited (Stenger et al., 1996).

Moreover, cases of recurrent leishmaniasis have been reported in patients receiving anti-TNF agents including infliximab, adalimumab and in very few cases, etanercept (Zanger et al., 2012; Guedes-Barbosa et al., 2013). The majority of recurrences has been observed in countries with an endemic reservoir including Mediterranean countries (Marcoval et al., 2017) or in holiday makers returning from endemic areas (Neumayr et al., 2013).

## Polarization Effector Mechanisms in Macrophages

### Macrophage Polarization

Peripheral monocytes and tissue macrophages display a remarkable plasticity *in vivo* (Gordon and Martinez, 2010; Varol et al., 2015). Depending on the nature and kinetics of a specific inflammatory response and a given cytokine environment, monocytes that enter tissues can rapidly develop into inflammatory effector M1 macrophages, or act as antigen-presenting cells (Murray, 2017). Subsequently, after the elimination of the inflammatory cues peripheral monocytes differentiate directly to M2 macrophages with a repair phenotype (M2 polarization) (Murray et al., 2014).

The definition of M1 and M2 macrophages as separate macrophage subpopulations is a concept based on *in vitro* experiments (Gordon, 2003). M1 and M2 macrophages represent the two extremes of a spectrum of physiological responses of macrophages to a very limited set of stimuli. The cytokine IFNγ which is characteristic of a T helper (Th) 1 response, polarized macrophages to a pro-inflammatory M1 phenotype. A similar outcome was also achieved by an engagement of TLRs (El Kasmi et al., 2008). In contrast, the Th2-type cytokines IL-4, IL-10, and/or IL-13 resulted in an alternative activation driven by the transcription factor STAT6 (Gordon and Martinez, 2010; Murray et al., 2014; Murray, 2017). The separate expression signatures of M1 and M2 macrophages were evident and could easily be distinguished (Martinez et al., 2013; Murray, 2017).

A major physiological difference in murine macrophages is the presence of the enzyme iNOS in M1 macrophages, which metabolizes L-arginine to nitric oxide (NO) and to reactive nitrogen species, and to citrulline which can be reused via the citrulline-NO cycle (Rath et al., 2014). High level expression of iNOS and the production of large amounts of NO provides M1 macrophages with the capacity to kill pathogens (Bogdan, 2015). Furthermore, M1 macrophages have been characterized by an expression of pro-inflammatory cytokines that aligns them with a Th1 response (TNF, type I IFN, IL-1β, IL-6, IL-12, IL-18, IL-23) (Murray et al., 2014; Murray, 2017).

In mouse models, M2 macrophages are characterized by expression of arginase (Arg1), an enzyme which hydrolyzes L-arginine to ornithine and urea. The arginase pathway facilitates the downstream synthesis pathways for polyamine and proline via ornithine, which M2 macrophages can utilize for tissue repair. Importantly, since Arg1 utilizes the same substrate as iNOS, Arg1 naturally limits L-arginine availability for NO synthesis by iNOS (Rath et al., 2014). M2 macrophages are aligned with the presence of Th2 cytokines such as IL-4, IL-13, and are associated with a response to parasitic diseases and late stages of an inflammatory immune response that supports wound healing and show in mouse models an expression of IL-10 and IL-6 (Murray et al., 2014). Indeed, arginine metabolic pathways cross-inhibit each other on the level of the respective arginine break-down products (Rath et al., 2014). This can have important effects for macrophage polarization and the immune response as will be discussed later (Rapovy et al., 2015; Schleicher et al., 2016).

While TNF is a major product of activated macrophages it also shows a strong influence on the activation and development of these cells. IFN-γ-induced macrophages express TNF in autocrine or paracrine manner (Mosser and Edwards, 2008) which synergistically enhances the expression of pro-inflammatory cytokines and microbicidal effector mechanisms (Bogdan et al., 1990; Liew et al., 1990a).

A single pulse of TNF produces an extended yet transient activation of the NF-κB pathway in macrophages (Hoffmann et al., 2002; Kalliolias and Ivashkiv, 2016) which, contingent on timing, results in induction of gene expression dependent on chromatin accessibility and availability of accessory proteins (Kalliolias and Ivashkiv, 2016). TNF activities are widespread but are predominantly pro-inflammatory in early stage immune responses (Kusnadi et al., 2019), serving to actively suppress M2 polarization in tumor associated macrophages and infection models (Kratochvill et al., 2015; Schleicher et al., 2016). The TNF-mediated restriction of M2 polarization is viewed to be an important contribution to an effective immune defense. Consequently, an absence of TNF or the blocking of its activities has consequences for the immune response to intracellular pathogens as macrophages are not only important effector cells but also targets of various species of intracellular pathogens. Given that macrophages are the primary targets for various species of intracellular pathogens, the following section discusses the importance of TNF on macrophage activation during such infections.

### Listeria monocytogenes

Nitric oxide is one of the central effector molecules for defense against *L. monocytogenes* infection (Beckerman et al., 1993; Leenen et al., 1994). For example, mice treated with iNOS inhibitors show an exacerbated infection in spleen and liver (Boockvar et al., 1994). Macrophages have been identified as the source of both NO and TNF in experimental *L. monocytogenes* infection. After infection monocytes are recruited to the spleen via CCR2 (Kurihara et al., 1997; Serbina et al., 2003) and differentiate into TNF/iNOS-producing CD11b^int^/CD11c^int^ dendritic cells (TipDCs). These cells are not required for *in vivo* T-cell priming but represent an effector arm of the innate immune system producing iNOS and TNF that elicit protective immune responses against *L. monocytogenes* (Serbina et al., 2003). Interestingly, iNOS-deficient mice are able to eliminate infections with a virulent *L. monocytogenes* strain despite a higher bacterial burden. However, mice with a double KO of iNOS in combination with gp91 phagocyte oxidase (phox) succumbed to *L. monocytogenes* infection, demonstrating that other effector mechanisms can compensate for an absence of NO (Shiloh et al., 1999). In the absence of TNF, TipDC-equivalent cells had an increased expression of Arg1 and harbored more bacteria (Li et al., 2017).

### Mycobacterium tuberculosis

Macrophages respond differently to infection by different genotypes of *M. tuberculosis*. However, a common signature is the expression of various amounts of iNOS, IL-1β, TNF, and IL-12 which corresponds to an M1 program (Chacon-Salinas et al., 2005). The concurrent exposure of macrophages to the pathogen and IFNγ was used as model to reveal the underlying transcriptomic changes, with these experiments demonstrating that iNOS and/or phagocyte oxidase (phox) had a central role in this reprogramming (Ehrt et al., 2001). This early stage M1 polarization was also demonstrated in alveolar macrophages as a component of granulomas in mice (Redente et al., 2010) and also in studies of human TB sufferers (Benoit et al., 2008). However, mycobacteria have developed strategies to avert a strong M1 response and the early secreted antigenic target protein 6 (ESAT-6) expressed by *M. tuberculosis* has been shown to significantly reduce the innate immune response significantly. Binding of ESAT-6 to TLR-2 prevented the downstream activation of NF-κB (Pathak et al., 2007).

A different line of research has shown Wnt6 signaling in granulomas induces arginase and downregulates TNF expression, driving a M2 polarization in a mouse model of *M. tuberculosis* infection (Schaale et al., 2013). This pathway and the entire Wnt network have been shown to be part of the conversation between pathogen and host and can be manipulated by *M. tuberculosis* (Villasenor et al., 2017). The alternative activation of macrophages is beneficial for the pathogen, since cellular L- arginine supply is limited and an activation of Arg-1 can rapidly deplete its levels, reducing the impact of iNOS via substrate competition. Interestingly, abrogation of arginase in iNOS-deficient mice under hypoxic conditions in granulomas resulted in increased bacterial burden and strong lung pathology pointing to an additional role of Arg-1 in immune regulation (Duque-Correa et al., 2014). However, macrophages under normal conditions can avoid undersupply of L-arginine by converting L-citrulline back to L-arginine, thus promoting their anti-mycobacterial activities (Rapovy et al., 2015). Notably, deficiency specifically in macrophage-expressed Arg1 resulted in a reduced *M. tuberculosis* burden and smaller cellular infiltrates in the lungs after aerosol infection (El Kasmi et al., 2008). Alternative activation has been demonstrated to result in reduced nitrosative stress of the pathogens (Kahnert et al., 2006). Likewise, IL-4 and IL-13 inhibited autophagy-induced killing of *M. tuberculosis* by macrophages (Harris et al., 2007).

### Leishmania major

Resistance to experimental *L. major* infections is driven by a strong Th1 response and an aligned M1 polarization of macrophages (Reiner and Locksley, 1995; Tomiotto-Pellissier et al., 2018). The expression of iNOS and the presence of NO have been shown to be critical for the elimination of *L. major* (Green et al., 1990; Liew et al., 1990b; Diefenbach et al., 1998). However, the role of TNF in this inflammatory response has been revisited and it was shown that TNF, while acting as pro-inflammatory mediator supporting M1 polarization, also actively blocked M2 polarization in *L. major* infected TNF^–/–^ mice (Schleicher et al., 2016). An analysis of skin lesions showed that TNF-deficient mice not only contained fewer CD11b^+^iNOS^+^ cells than did WT lesions but additionally almost all iNOS^+^ cells co-expressed Arg1. This occurred only in around 50% of WT CD11b^+^iNOS^+^ cells. This overexpression of Arg1 in the absence of TNF resulted in a lack of tyrosine nitration in the lesion and the draining lymph nodes, which caused by a strongly reduced activity of NO in the tissue despite a presence of iNOS (Schleicher et al., 2016). Independent of the potential competition with NO during the acute stage, Arg1 has recently been reported to be dispensable for the resolution of cutaneous leishmaniasis (Paduch et al., 2019).

## Concluding Remarks

While there is now broad agreement about the development and activation pathways of monocytes and macrophages, it is increasingly clear that we are not looking at one relatively homogenous population but a continuum of very different developmental states (Hume, 2015; Guilliams et al., 2018). Macrophage differentiation responds dynamically to environmental changes *in vivo* even under so called steady state conditions (Hume, 2015). Under inflammatory conditions this situation becomes even more fluid. Antigenic stimuli such as pathogens are highly dynamic and cytokines do not exist in isolation in inflamed tissues. Because the transcriptome of macrophages displays such high plasticity it has been accepted that various responses to environmental signals occur and consequently, various molecule signatures can co-exist and change rapidly. The example of a color wheel has been used where every combination of colors is possible (Mosser and Edwards, 2008) and this extension from polarization to spectrum model is now well supported experimentally (Xue et al., 2014).

As a consequence, the notion to define myeloid cell populations by only a few marker molecules has been challenged. Identifying combinations of key marker molecules that accurately characterize macrophage subpopulations and related dendritic cells in the *in vivo* context has been a major goal, but it is not fully supported by the transcriptional analysis of signature molecules (Gautier et al., 2012; Miller et al., 2012; Hume, 2015). Rather, new approaches using single cell transcriptomics are now revealing previously hidden insights in the heterogeneity of myeloid cell populations (Shalek et al., 2013, 2014) and can be used to better understand the behavior of tissue specific cell populations such as microglia (Li et al., 2019) or the cell specific changes that follow the conversation between immune cell and pathogen and that underlie the immune response (Avraham et al., 2015). Therefore, an analysis of *in vivo* models at a single cell level represents the ultimate challenge and the understanding of the regulation of transcriptional changes in response to a dynamic and complex environment is still in its infancy.

For many years, TNF has been acknowledged as an important pro-inflammatory cytokine that plays a clearly important yet still only vaguely defined role in the immune response. A puzzling observation has been the dominant part it plays in response to intracellular infections by bacteria and parasites where the humoral response is subservient. Recently, a new role of TNF in the biology of macrophages was demonstrated (Schleicher et al., 2016) and the importance of effective polarization was emphasized. A dysregulated macrophage polarization that leads to an ill-timed accumulation of M2 macrophages and as consequence a lack of proinflammatory mediators and effector molecules due to either absence or suppression of TNF could be an explanation for the increased sensitivity to this category of pathogens. Further research will be needed to gage the extent how an effective and timely macrophage polarization reigns in the early spread of pathogens and supports the general immune response.

## Author Contributions

XLi: original draft preparation. HK and XLiu: review, editing, and supervision. XLi and XLiu: funding acquisition. All authors contributed to the article and approved the submitted version.

## Conflict of Interest

The authors declare that the research was conducted in the absence of any commercial or financial relationships that could be construed as a potential conflict of interest.
